# Gastrointestinal Tolerance and Protein Absorption Markers with a New Peptide Enteral Formula Compared to a Standard Intact Protein Enteral Formula in Critically Ill Patients

**DOI:** 10.3390/nu13072362

**Published:** 2021-07-10

**Authors:** Ione de Brito-Ashurst, Marianne Klebach, Eleni Tsompanaki, Sundeep Kaul, Peter van Horssen, Zandrie Hofman

**Affiliations:** 1 Royal Brompton and Harefield NHS Foundation Trust, Royal Brompton Hospital, Sydney Street, London SW3 6NP, UK; Ione.Ashurst@rmh.nhs.uk (I.d.B.-A.); elena.tsompanaki@nhs.net (E.T.); sunnykaul@aol.com (S.K.); 2 The Royal Marsden NHS Foundation Trust, Fulham Road, London SW3 6JJ, UK; 3 Danone Nutricia Research, Utrecht University, 3584 CT Utrecht, The Netherlands; Peter.VAN-HORSSEN@danone.com (P.v.H.); zandrie.hofman@danone.com (Z.H.); 4 Imperial College Healthcare NHS Trust, St Mary’s Hospital, London W2 1NY, UK

**Keywords:** hydrolyzed protein, polymeric formula, ICU, gastrointestinal tolerance, nutritional guidelines, protein absorption

## Abstract

The aim of this exploratory study was to investigate gastrointestinal tolerance and protein absorption markers with a new enteral peptide formula (PF) compared to an isocaloric enteral intact protein standard formula (SF) containing the same amount of protein in ICU patients. Patients admitted to a cardio-thoracic intensive care unit expected to receive tube feeding for ≥5 days were randomized to receive either PF (1.5 kcal/mL) or SF in a double-blind manner for ≤14 days. Twenty-six patients were randomized (13 SF and 13 PF) and 23 (12 SF and 11 PF) completed at least 5 days of product administration. There were no statistically significant differences between the feeds during the first 5 days of intervention for diarrhea (SF:3 (23%); PF:5 (39%), *p* = 0.388), vomiting (SF:1 (8%); PF:2 (15%), *p* = 0.549), constipation (SF:7 (54%), PF:3 (23%), *p* = 0.115), and high gastric residual volume (>500 mL: SF:1 (8%); PF: 2 (15%), *p* = 0.535). There were no differences in plasma amino acids or urinary markers of protein absorption and metabolism. In conclusion, no major differences were found in tolerability and protein absorption markers between the standard intact protein formula and the peptide formula.

## 1. Introduction

Standard intact protein tube feed formula is generally recommended as a first-line treatment for critically ill patients, whilst for those presenting with severe malabsorption, peptide or semi-elemental feeds are suggested [1,2,3,4,5,6]. Nutrient malabsorption is associated with gastrointestinal symptoms, such as diarrhea, bloating, and abdominal pain [7,8]. These complications cause discomfort and more importantly can result in nutrient losses and decreased nutritional intake, which is detrimental to patient recovery [9].

Peptide formulas are based on hydrolyzed protein, requiring less enzymatic breakdown before entering the bloodstream, which could potentially improve absorption and reduce gastrointestinal problems [10].

While there is a lack of clear evidence supporting the general use of peptide-based formulas it is recognized that some patients may benefit from changing to a peptide formula. [2,3,4,5]. For example, Heyland and colleagues proposed a feeding protocol for critically ill patients starting with peptide instead of intact protein feeds [11]. Additionally, ASPEN guidelines suggest considering small-peptide formulas as a second-line treatment in patients with persistent diarrhea and suspected malabsorption or lack of response to fiber [4]. Further research is needed to define the clinical situations in which peptide-based formulas should be prescribed. 

Beyond the potential positive effects on gastrointestinal intolerance, benefits of hydrolyzed feeds on protein absorption and nitrogen balance have been suggested [12,13]. This could be relevant for critically ill patients because of their decreased pancreatic secretory capacity [14]. However, studies in this population, comparing parameters of protein absorption and metabolism between intact protein and peptide feeds, are scarce and outcomes are difficult to translate to a typical critically patient. Moreover, studies were performed more than 20 year ago with enteral formulas that are no longer available [13,15].

Irrespective of the type of enteral feed used, most critically ill patients receive less protein than prescribed [16]. Meeting nutritional targets is a challenge and, moreover, available tube-feeding formulas often contain a relatively low proportion of protein. Recognizing that high-protein feeds are paramount to deliver optimal nutrition, a new peptide formula was developed to offer the advantage of peptides with a higher protein content.

The current exploratory study was devised to gain insights in gastrointestinal tolerance and various indicators of protein absorption and utilization with a new peptide formula, compared to a standard intact protein formula with the same protein content in a population of critically ill patients.

## 2. Materials and Methods

### 2.1. Study Design

This was a randomized, controlled, double-blind, and parallel-group study. The study was carried out in a cardiothoracic critical care setting at a tertiary hospital center across two sites in London, United Kingdom. 

The study protocol was approved by the institutional review board and registered under Dutch trials, registered with the identifier: NL5798 (http://www.trialregister.nl/trialreg (accessed on 9 July 2021)).

Study procedures were performed in accordance with the Declaration of Helsinki ethical principles for medical research involving human subjects. Written informed consent was obtained from patients or their next of kin.

The study population comprised mechanically ventilated patients (age ≥ 18 years) admitted to the ICU. Eligible patients were those expected to require EN starting within 48 h after ICU admission, and continuing for >5 days. Exclusion criteria were a sequential organ failure assessment (SOFA) score of >12, any contraindication to tube nutrition, and abnormalities in the gastrointestinal tract that could impair function. A complete list of inclusion and exclusion criteria is provided in the Supplementary Materials (Table S1).

### 2.2. Intervention

Patients were randomized within 48 h of admission, to receive either the new peptide formula (PF) or the standard formula (SF) until the end of the ICU stay or for a maximum period of 14 days. Permuted block randomization for either the test or control group was stratified per study location. The randomization sequence was computer-generated by a blinded statistician not involved in data collection or analysis. The randomization code was broken after database lock to enable calculations of the study outcomes. The ready-to-use study products had identical packaging. Patients, investigators, and medical staff were blinded to treatment allocation.

The study products were isocaloric (150 kcal/100 mL) and contained the same amount of protein (7.5 g protein/100 mL). The PF was based on hydrolyzed whey protein with 51% of energy from carbohydrates and 29% from fat. The SF was a commercially available tube feed, based on intact proteins (P4 protein blend: casein, whey, soy, pea; Nutrison Protein Plus Energy, Nutricia, Zoetermeer, The Netherlands). More details on the product compositions can be found in the Supplementary Materials (Table S2).

The study products were administered through a nasogastric tube. Although the sites could adhere to their own feeding protocol and targets, it was recommended to start EN at a rate of 20 mL/h, gradually increasing until the target of 25 kcal/kg bodyweight (BW)/day was achieved. For patients with a BMI > 30 kg/m^2^, adjusted BMI was used to define the target volume, where adjusted body weight (kg) was calculated as: 30 × (height in meters^2^). These recommended target volumes were used to define the number of days for which the target was not reached. Supplementary feeding with parenteral nutrition was allowed, if necessary.

### 2.3. Data Collection

Demographic and medical information including age, sex, height, weight, acute physiology and chronic health evaluation (APACHE) II score, SOFA score, and admission category (medical, surgical, or trauma) were collected at baseline.

The volume of administered study product was recorded daily until ICU discharge or day 14 of intervention. Reasons were recorded for each day that the target intake was not met (start-up period, gastrointestinal problems, medical procedures, intake via other routes, and “other” reasons). 

Gastrointestinal tolerance parameters were recorded on a daily basis by use of a questionnaire during the intervention period, including defecation frequency, defecation consistency (according to Bristol Stool Form Scale), gastric residual volume (GRV), and vomiting. 

At baseline and day 5, blood samples for determination of serum amino acid concentration were collected in serum separating tubes. The serum samples were stored at −80 °C for analyses at a central laboratory. The content of the individual amino acids was determined in the supernatant by ultra-fast liquid chromatography using a pre-column derivatization with o-phthaldialdehyde and fluorimetry as detection. The samples were analyzed in one batch at the end of the study. 

At day 5, urine was collected for 24 h. Three samples of 9 mL were stored at −80 °C until analysis. Total nitrogen was analyzed by measuring urea by enzymatic reaction. P-cresol and phenol were analyzed by high-performance liquid chromatography (HPLC) with fluorometric detection and creatinine by enzymatic reaction and colorimetric detection of the final product after the reaction. 3-Methylhistidine was analyzed by HPLC with mass spectrometry detection.

Adverse events that were probably, possibly, or definitely related to the study product, in the judgment of the treating physician, or not related to the underlying condition were recorded. All serious adverse events (SAEs) were recorded. Adverse event recording continued until the end of the follow-up period (28 days). SOFA scores and samples for analyses of liver and renal function (alkaline phosphatase, ALT, AST, γ-GT, ammonia, creatinine, BUN, and cystatin-C) were collected at baseline, day 5, and at day 14, or on the day of ICU discharge. 

### 2.4. Outcomes

The main outcome parameters in this study were measures of gastrointestinal tolerance including incidence of diarrhea, constipation, vomiting, and high GRV (250 and 500 mL). The incidence of diarrhea was assessed using the daily defecation score (DDS), which is the sum of consistency scores for every evacuation per day. Diarrhea was defined as a DDS of > 15 for at least 1 day or a DDS between 6 and 15 for at least 2 consecutive days (previously described in [17]). Constipation was defined as no bowel evacuation for 72 h. 

Study product intake is reported as kcal/kg BW received from study product. The proportion of days at which the target of 25 kcal/kg BW was not reached is reported both as average per subject and as a percentage of all intervention days. Reasons for not reaching the target are reported as percentage of all intervention days.

The focus of the study was on the first 5 days of the intervention period because most measurements were performed at day 5, and it was expected that patient numbers requiring tube feeding would decrease from day 5 onwards. Therefore, the reported data on nutritional intake and tolerance parameters represent the first 5 days of intervention unless otherwise indicated. Data were included until study product intake stopped permanently. 

Outcome parameters at day 5 included measurements of serum amino acid concentrations, protein fermentation in the colon measured by urinary p-cresol (mg/24 h) and urinary phenol (mg/24 h) [18,19], whole body protein loss by urinary nitrogen excretion (g/24 h), muscle protein breakdown by 3-methylhistidine (mmol/24 h) and creatinine (mmol/24 h) [20]. 

Safety assessments included patient medical history, medication use, blood safety parameters (baseline, day 5 and day 14, or on the day of ICU discharge), and adverse events throughout the intervention period and at follow-up (day 28). 

Adverse events and blood safety parameters were evaluated in detail per patient in a blinded fashion. In addition to the individual evaluation, blood safety parameters were compared between study groups by use of descriptive statistics including the number of patients with values: (1) outside the normal range, (2) outside the normal range and clinically relevant, (3) clinically relevant and related to the underlying condition. In addition, statistical comparisons were performed for the measured values between and within study groups. See Supplementary Materials Table S5 for an example of the summary statics used.

An independent data monitoring committee (DMC) performed a safety review after 15 patients had completed the study.

### 2.5. Statistical Analysis

All analyses were performed on the all-subjects treated data set. Thirty patients were planned to be included. No sample size calculation was performed because the goal of this exploratory study was to gain insights in gastrointestinal tolerance and protein absorption which cannot be well defined by a single symptom or value [21]. Therefore, it was decided to evaluate tolerance and protein absorption based on the profile over several parameters. 

For continuous outcome parameters, mean ± standard deviation (SD) or median interquartile range (IQR), for skewed distributed data, are reported unless otherwise specified. For incidences, n (%) are reported and compared across the intervention groups using Cochran–Mantel–Haenszel (stratifying for center).

Continuous outcome parameters were analyzed using a 2-way analysis of variance (ANOVA), including the factors center and intervention group. For the comparison of plasma amino acids at day 5, baseline was included as an additional factor. When the normality assumption was not satisfied, nonparametric Van Elteren tests (stratified Wilcoxon–Mann–Whitney test) were performed to compare the intervention groups.

For all outcome parameters, 2-sided *p* values < 0.05 were considered statistically significant, without corrections for multiple testing. Analyses were performed with SAS software, version 9.4 (SAS Institute Inc, Cary, NC, USA).

## 3. Results

Patients were recruited between 8 December 2016 and 21 September 2017. In total, 26 patients were randomized, 13 to the PF group and 13 to the SF group. All 26 randomized patients were included in the statistical analyses. Ten patients in the PF group and 11 in the SF group completed the first 5 days of the study. Two patients in the PF group and 6 in the SF group completed the full 2-week intervention period. An overview of the number of patients per intervention day can be found in the Supplementary Materials, Table S6. Patients with a shorter period of study product administration were either discharged from the ICU, died, or stopped receiving study product. Reasons for stopping study product in the PF group included change to normal hospital diet, change to other enteral feed due to presumed need for concentrated feed or fiber, and change to PN, whereas use of other enteral feed, normal hospital diet, and high aspirates volumes were reasons for stopping in the SF group.

### 3.1. Baseline Characteristics

The groups were well balanced for all baseline characteristics except for some differences in the scores indicating disease severity; the median APACHE II score was higher in the PF group than in the SF group, translating into differences in predicted mortality, whereas, the median SOFA score was higher in the SF group than the PF group. Table 1.

### 3.2. Study Product Intake and Gastrointestinal Tolerance

Median daily energy intake from study product was 14.6 (IQR: 11.8−15.9) kcal/kg BW with PF versus 16.7 (IQR: 12.2−19.6) kcal/kg BW with SF (*p* = 0.109). The reported reasons for not reaching the caloric target in the first 5 days are shown in Table 2. See Supplementary Materials, Table S4 for the reasons per intervention day. Almost half of the reasons were in the predefined category “other”, most commonly; tube pulled out, tracheostomy, waiting for surgery, and target according to local protocol met. 

There were no statistically significant differences in tolerance parameters (diarrhea, constipation, vomiting, and incidence of high GRV) in the first 5 days (Table 3).

Median time to first defecation was 41 (IQR: 13−66) in the PF group versus 36 (IQR: 6−77) h in the SF group. For the total intervention period, similar outcomes were found in both groups, except for a statistically significantly lower incidence of constipation (3 versus 9 patients, *p* = 0.023) and a non-significantly lower study product intake in the PF group (12.7 (11.9−17.0) versus 18.1 (13.4−20.7) kcal/kg BW, *p* = 0.051). 

### 3.3. Plasma Amino Acids and Urine Parameters of Protein Metabolism

**Figure 1:**
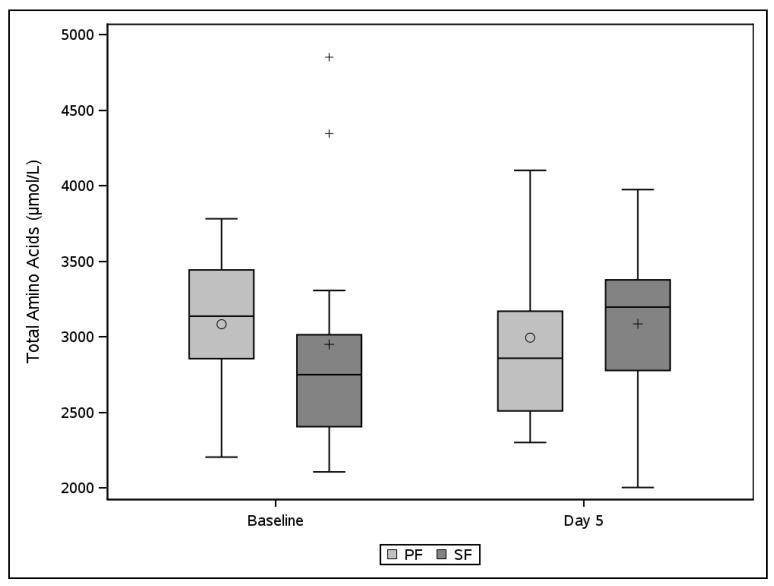
Plasma amino acid concentration. Total plasma amino acid concentration (µmol/L) at baseline and day 5 comparing the peptide formula (PF) and the standard formula (SF) groups. Baseline: SF; *n* = 12, PF; *n* = 11. Day 5: SF; *N* = 11 and PF *n* = 10. Box plot interpretation: ○ or + average value, −: median, rectangle bottom: quartile 1 cut point (25th percentile), rectangle upper: quartile 3 cut point (75th percentile); 0 or +: outliers more than 1.5 times IQR above quartile 3 or below quartile 1, T: highest or lowest level not being an outlier. Statistical analyses based on a 2-way analysis of variance (ANOVA) with treatment and center as factors.

Serum concentrations of total amino acids (TAA) did not differ statistically significantly between PF and SF, either at baseline (*p* = 0.664) or day 5 (*p* = 0.565). In addition, TAA serum concentrations did not change statistically significantly over time (*p* = 0.565) (Figure 1).

No statistically significant differences were found between groups for any of the individual amino acids. Urinary p-cresol and urinary phenol did not differ statistically significantly between PF and SF on day 5 (*p* = 0.520 versus 0.317). Urinary total nitrogen, urinary creatinine, and urinary 3-methylhistidine did not differ statistically significantly between PF and SF on day 5 (Table 4).

### 3.4. Clinical Outcome Parameters

The clinical outcome parameters including mortality, SOFA score, and durations of ICU stay, hospital stay, and mechanical ventilation did not show any statistically significant differences between the PF and SF groups (Table 5).

### 3.5. Safety Parameters

A total of 17 adverse events were reported in 10 of the 26 patients included in the study (38.5%): 8 events in 6 patients (46.2%) in the PF group and 9 events in 4 patients (30.8%) in the SF group. 

In total, 14 SAEs were reported in 9 of 26 patients (34.6%). None of the serious adverse events were related to the study product. 

There were 5 gastrointestinal events in 4 patients: 2 events in 2 patients in the PF group (diarrhea and intestinal ischemia) and 3 events in 2 patients (vomiting and intestinal ischemia) in the SF group. Two mild adverse events, diarrhea (PF group) and vomiting (SF group) were rated as “possibly” and “probably” related to the study product, respectively. 

There were two cases of intestinal ischemia in the standard formula group (occurring 8 vs 14 days after first day of study product intake) and one in the control group (occurring 11 days after start study product intake). All 3 cases were unlikely related to the study products as judged by the investigator.

See Supplementary Material, Table S3 for adverse event details.

The recorded laboratory measurements showed an erratic pattern during the course of the study and were evaluated on an individual basis. All clinically relevant measurements outside the normal ranges were considered by the investigator to be related to the underlying condition and not the study product. 

There were no statistically significant differences between groups in laboratory parameters measuring changes from baseline to day 5, day 14, or the end of study (day of discharge if discharged before day 14), except for BUN, which showed a statistically significant difference in the change from baseline to the end of study with a higher mean value in the PF group than the SF group (*p* = 0.021) but not at day 14 (*p* = 0.200) and ASAT, which showed a significantly greater decrease from baseline to day 14 in the SF group than the PF group (*p* = 0.020) but not at end of study (*p* = 0.768).

After evaluation of the semi-blinded interim analysis data (SAEs and mortality) the DMC recommended continuing the study as planned.

## 4. Discussion

The purpose of this study was to explore tolerance and markers of protein absorption with a new peptide formula compared to a standard EN feed in a complex cardiothoracic ICU patient population. 

The results showed no statistically significant differences in gastrointestinal complications, plasma amino acids and urine markers of protein metabolism between the study groups for the first 5 days of intervention.

Study product intake was not statistically significantly different between groups and generally far below target, comparable to the caloric intake reported in other nutrition studies performed in ICU patients [22,23]. For the total intervention period, the caloric intake tended to be lower in the PF group. This difference seems to be caused mainly by low intake related to medical procedures and practical issues, which were frequently reported. Intolerance was only reported in a minority of cases. This is in line with previous reports indicating that feeding interruptions are mostly procedure-related [24]. 

There were no differences in diarrhea, high GRV, vomiting, and constipation in the first five days. The incidence of constipation was higher in the SF group only when the available data for the total intervention period were included. Although this may indicate a potential effect of the PF on the reduction of constipation, the interpretation of these data is confounded by the fact that in the SF group the average duration of intervention was longer, and influenced by the frequent use of opioid analgesics in both intervention groups. Based on the hypothesized effect of hydrolyzed protein on increasing the absorption of nutrients, the opposite effect is more likely because higher amounts of unabsorbed nutrients in the bowel, are commonly associated with diarrhea [8,25,26]. However, in the current study, no differences were found between groups for this parameter. The available literature on the effects of peptide feeds compared to intact protein feeds on diarrhea show conflicting results Two small pilot studies performed around the 1990s reported a lower incidence of diarrhea [27,28], but these outcomes were not confirmed in two subsequent, larger RCTs, which showed no differences in diarrhea in a similar ICU population [15,29]. Our study results are in line with the two larger RCTs that did not show a difference in diarrhea incidence between a peptide and standard formula.

Some data in non-critically ill patients suggest that PF may enhance gastric emptying rate [30,31], which may result in a lower incidence of vomiting and high gastric retention. Based on the measurements of upper gastrointestinal symptoms and plasma amino acids, there were no indications of such an effect. The difference found in previous studies could have been specific for the comparison with a casein-based standard formula, which could decrease gastric emptying because of coagulation of the protein in the stomach [32].

Independent of gastrointestinal tolerance and nutritional intake, also a direct effect of peptide feeds on protein bioavailability and nutritional status has been suggested [13,28]. Although enhanced protein absorption could be very relevant in critically ill patients in whom endocrine pancreatic insufficiency is common, the markers of protein absorption measured in the current study do not indicate a difference between the feeds. This may indicate that no significant malabsorption was present in this population or, alternatively, that intestinal digestion of proteins was not the limiting factor in protein absorption [8]. The indicators of protein absorption measured in the current study were plasma amino acid levels and urinary markers of protein fermentation in the colon. Plasma amino acid seem to be fairly representative of dietary amino acid bioavailability levels despite influence of endogenous protein breakdown [33]. In addition, it has been shown that a steady state is reached during continuous feeding [34]. Nevertheless, the single measurement done at day 5 of intervention remains a rough indicator of dietary protein bioavailability. To gain additional insights into protein absorption, we measured urinary p-cresol and phenol as surrogate markers of the amount of unabsorbed protein reaching the colon for which the relevance was demonstrated previously [18,19]. Our study showed no differences between SF and PF for these parameters. However, a high interindividual variation was observed. Therefore, the validity and meaning of these outcomes should be further investigated.

Lastly, urine parameters indicative of protein utilization were measured, including urinary nitrogen, 3-methylhistidine, and creatinine. These parameters were similar between groups, which is in line with other studies that showed no difference in total body nitrogen balance or whole-body protein utilization with peptide feeds [15,35].

Several limitations of the current study need to be recognized. The population chosen for this study is one of the most vulnerable patient groups expected to receive the PF. With our population being sicker the typical ICU population. This may limit generalizability of the study findings. High heterogeneity in their medical condition and treatments need to be considered when interpreting the data. Additionally, variation in the duration of intervention, because of death, discharge, and supplemental use of other sources of nutrition, further complicates the comparison between groups. To lessen the influence of variation in these factors. this study focused on the first 5 days of intervention. 

A statistically non-significant higher APACHE score was reported for the PF at baseline which may have influenced the outcome parameters. However, the possible impact on the outcomes is difficult to judge, especially because the SOFA score at baseline was higher in the SF group.

Overall, the outcomes of this study should be interpreted with caution because of the small sample size. No strong conclusions should be drawn from these data, which should rather be seen as hypothesis-generating. 

Despite these limitations, the results of this study add interesting data to the very limited amount of information from randomized controlled trials comparing hydrolyzed with intact protein formula providing the same amount of protein. 

## 5. Conclusions

In this study the new peptide formula was found to have a tolerance profile comparable to standard tube feeding. This suggests that the new peptide feed is suitable to initiate tube feeding in critically ill patients upon ICU entry, but does not seem to offer benefits compared tube feeding initiation with a standard tube feed on tolerance or protein absorption. Potential advantages in patients with proven malabsorption or persistent intolerance to standard feeds remains to be investigated [36]. For example, a well-designed and adequately powered trial to investigate the effect of PF on diarrhea in patients who do not tolerate standard tube feeds would be of interest. Until more data are available, nutrition experts should rely on their expertise and clinical judgment to select the best feed for their patients.

## Figures and Tables

**Table 1:** Baseline characteristics.

		SF (*N* = 13)	PF (*N* = 13)	*p*-Value ^1^
Sex (male)	*n* (%)	6 (46.2%)	8 (61.5%)	0.695
Age (years)	Mean (SD)	54.5 (16.8)	56.5 (18.8)	0.786
BMI (kg/m^2^)	Mean (SD)	27.40 (4.98)	29.25 (5.63)	0.383
Admission diagnosis				1.000
Medical	*n* (%)	8 (61.5%)	9 (69.2%)	
Surgical non-trauma	*n* (%)	4 (30.8%)	4 (30.8%)	
Trauma non-surgical	*n* (%)	1 (7.7%)	0 (0.0%)	
SOFA score	Median [IQR]	10 (8−11)	7 (6−11)	0.286
APACHE II score	Median [IQR]	21 (16−24)	26 (18−28)	0.353
Predicted mortality (%)	Mean (SD)	37.55 (19.97)	46.58 (22.86)	0.294
Adjusted predicted mortality (%)	Mean (SD)	23.4 (17.5)	35.8 (26.0)	0.164

¹ *p*-value for numeric variables is based on T-test, for categorical variables on Fisher’s exact test.

**Table 2:** Listing of reasons for target not reached.

	Total (*N* = 26)	SF (*N* = 13)	PF (*N* = 13)
Total no. days	123	62	61
Total % of days target not reached	86.2%	82.3%	90.2%
Reported reasons ^1^			
Start-up period, *n* (%)	23.6%	22.6%	24.6%
Symptoms of intolerance, *n* (%)	7.3%	4.8%	9.8%
Medical investigation, *n* (%)	12.2%	9.7%	14.8%
Energy intake other routes, *n* (%)	10.6%	4.8%	16.4%
Other ^2^, *n* (%)	42.3%	45.2%	39.3%

^1^ Reasons are based on daily recording with predefined categories. Reported as % of total number of days. More than one reason could per reported per day. ^2^ Other reasons included: tube pulled out, tracheostomy, waiting for surgery, and target according to local protocol met.

**Table 3:** Incidence of gastrointestinal tolerance symptoms ^1^.

		SF (*N* = 13)	PF (*N* = 13)	*p* Value ^4^
Diarrhea ^2^	*n* (%)	3 (23.1%)	5 (38.5%)	0.388
Constipation ^3^	*n* (%)	7 (53.8%)	3 (23.1%)	0.115
Vomiting	*n* (%)	1 (7.7%)	2 (15.4%)	0.549
GRV > 250 mL	*n* (%)	5 (38.5%)	4 (30.8%)	0.691
GRV > 500 mL	*n* (%)	1 (7.7%)	2 (15.4%)	0.535

^1^ Incidence is defined as the number (%) of patients who experienced at least one event during the first 5 days of the intervention period. ^2^ Diarrhea is defined as DDS > 15 for at least 1 day and/or a DDS ≥6 for at least 2 consecutive days. ^3^ Constipation is defined as no bowel evacuation within 72 h. ^4^ *p* value derived from Cochran–Mantel–Haenszel (stratifying for center). GRV: Gastric residual volume.

**Table 4:** Urinary parameters.

		SF (N = 13)	PF (N = 13)	*p* Value ^1^
		*n* = 8	*n* = 9	
p-Cresol (mg/24 h)	Median (Q1−Q3)	21 (10−54)	48 (6−151)	0.520
Phenol (mg/24 h)	Median (Q1−Q3)	1 (0−22)	0 (0−2)	0.317
Total nitrogen (g/24 h)	Median (Q1−Q3)	18 (9−20)	15 (5−23)	0.981
Nitrogen balance (g)	Median (Q1−Q3)	−46 (−56−13)	−38 (−57−49)	0.548
Creatinine (mmol/24 h)	Median (Q1−Q3)	5 (3−9)	4 (2−8)	0.924
3-Methylhistidine (µmol/24 h)	Median (Q1−Q3)	268 (106−345)	262 (32−390)	0.775

^1^ Based on van Elteren test (stratified for center).

**Table 5:** Clinical outcome parameters.

		SF (*N* = 13)	PF (*N* = 13)	*p* Value
Mortality rates (28 days)	*n* (%)	4 (30.8%)	4 (30.8%)	0.930 ^1^
Duration of ICU stay (days)	Mean (SD)	12.7 (6.0)	14.5 (8.2)	0.539 ^2^
Duration of hospital stay (days)	Median (Q1−Q3)	16 (10−29)	29 (17−29)	0.361 ^3^
Duration of first ventilation period (days)	Median (Q1−Q3)	10 (8−14)	11 (6−14)	0.618 ^3^
SOFA score (at Day 5)	Median (Q1−Q3)	7 (5−10)	6 (5−10)	0.713 ^3^

^1^ Time to death tested with Cox proportional hazards regression analysis with study product and stratification factor. ^2^ Based on a 2-way analysis of variance (ANOVA) with treatment and center as factors. ^3^ Based on van Elteren test (stratified for center).

## Supplementary Materials

The following are available online at https://www.mdpi.com/article/10.3390/nu13072362/s1. Tables S1: In- and exclusion criteria. Tables S2: Study product composition. Tables S3a: Individual data listing of subjects with at least one adverse event—Peptide Formula. Tables S3b: Individual data listing of subjects with at least one adverse event—Standard Formula. Tables S4: Number of patients that did not reach target volume tube feed including reason for each single day. Tables S5: Summary statistics for ASAT (U/L) day 5. Tables S6: Number of patients (n) per intervention day.
