# Supplementary material for: Gastrointestinal Tolerance and Protein Absorption Markers with a New Peptide Enteral Formula Compared to a Standard Intact Protein Enteral Formula in Critically Ill Patients
Source: Nutrients. 2021 Jul 10;13(7):2362. doi: 10.3390/nu13072362 (PMC8308653; doi:10.3390/nu13072362)
Supplement: Supplementary file 1 [file nutrients-13-02362-s001.zip › nutrients-1262950-supplementary.pdf]

Table S1

In- and exclusion criteria

Inclusion criteria

1. Age ≥ 18 years
2. Admitted to the ICU
3. Expected to require enteral feeding with the study product on the ICU for at least 5 days
4. Aim to reach nutritional target by study product within 3 days
5. Start of study product administration within 48 hours after ICU admission
6. Written informed consent of subject or legal representative

Exclusion criteria

1. Requiring other tube feed for medical reason
2. Having any contraindication to receive tube feed such as severe shock, presence of partial or complete mechanical bowel obstruction, intestinal ischemia or intestinal infarction
3. Known history of allergy or intolerance to the study product components (test or control product), such as allergy for cow's milk protein, soy or pea protein, and patients with galactosemia
4. Abnormalities in gastrointestinal tract which may impact gastrointestinal function, e.g. short bowel syndrome - defined as entire length of small bowel totaling 200 cm or less, ulcerative colitis or Crohn's disease, or any form of enterostomy
5. Known chronic severe renal failure
6. Known chronic severe hepatic failure
7. Gastrointestinal tract, abdominal, or bariatric surgery within 72 hours before starting intake of study product or expected in the next 5 days after starting study product intake
8. History of chronic pancreatitis or acute pancreatitis
9. SOFA score >12 from admission to the ICU until 24 hours after admission or until randomization in case of randomization before 24 hours after admission
10. Being pregnant
11. Participating in another clinical intervention trial

Table S2

Study product composition

	SF		PF	
	Per 100 ml	% of energy	Per 100 ml	% of energy
Energy	150 kcal		150 kcal	
Protein	7.5 g	20%	7.5 g	20%
-whey<br>-casein<br>-pea<br>-soy	2.6 g<br>1.9 g<br>1.5 g<br>1.5 g		7.5 g (whey hydrolysate)	
Carbohydrate	16.9 g	45%	19.0 g	51%
Fat	6.8 g	35%	4.9 g	29%
-MCT	0.9 g		3.3 g	
Fibre	-	-	-	-

Table S3a

Individual data listing of subjects with at least one adverse event - Peptide Formula

**Subject ID**	**Verbatim**	**Preferred term**	**Product start date [yyyy-mm-dd]**	**AE start date [yyyy-mm-dd]**	**AE end date [yyyy-mm-dd]**	**Duration of AE¹**	**Severity**	**Relationship to study product**	**SAE? [Yes/No]**
GB-301-003	Diarrhoea	Diarrhoea	2017-01-26	2017-01-31	Ongoing		MILD	POSSIBLY	No
GB-301-005	Ischaemic Bowel (Pneumatosis Intenstinalis)	Intestinal ischaemia	2017-02-10	2017-02-21	2017-03-01	9	SEVERE	UNLIKELY	Yes
GB-301-011	Septic shock with multi-organ failure	Septic shock	2017-05-27	2017-06-15	2017-06-15	1	SEVERE	UNLIKELY	Yes
	Septic shock with multi-organ failure	Multiple organ dysfunction syndrome	2017-05-27	2017-06-15	2017-06-15	1	SEVERE	UNLIKELY	Yes
GB-301-014	high gastric residual volume (1X 380 ml, 1X 540 ml, 1X 210 ml)	Gastric residual assessment	2017-07-05	2017-07-09	2017-07-09	1	MILD	UNLIKELY	No
	Multi-organ failure	Multiple organ dysfunction syndrome	2017-07-05	2017-07-15	2017-07-15	1	SEVERE	UNLIKELY	Yes
GB-302-001	Multi organ failure	Multiple organ dysfunction syndrome	2016-12-21	2016-12-23	2016-12-27	5	SEVERE	NOT RELATED	Yes
GB-302-004	Very severe global hypoxic encephalopathy	Hypoxic-ischaemic encephalopathy	2017-03-23	2017-04-04	2017-04-04	1	SEVERE	NOT RELATED	Yes

Table S3b

Individual data listing of subjects with at least one adverse event - Standard Formula

Subject ID	Verbatim	PT	Product start date	AE start date	AE end date	Duration of AE[1]	Severity	Relationship to study product	SAE? [Yes/No]
GB-301-001	100 ml vomiting	Vomiting	2016-12-08	2016-12-17	2016-12-17	1	MILD	PROBABLY	No
	Sepsis	Sepsis	2016-12-08	2016-12-22	2016-12-23	2	SEVERE	UNLIKELY	Yes
	Bowel ischaemia, which was triggered by Granulomatosis with polyangiitis	Intestinal ischaemia	2016-12-08	2016-12-22	Ongoing		SEVERE	UNLIKELY	Yes
GB-301-010	Acute Myocardial Infraction	Acute myocardial infarction	2017-04-06	2017-04-08	Ongoing		SEVERE	NOT RELATED	Yes
	Cardiogenic shock (as a result of	Cardiogenic shock	2017-04-06	2017-04-08	2017-04-08	1	SEVERE	NOT RELATED	Yes
GB-302-003	Multi-organ failure	Multiple organ dysfunction syndrome	2017-01-25	2017-02-01	2017-02-01	1	SEVERE	NOT RELATED	Yes
GB-302-007	Gut ischaemia,	Intestinal ischaemia	2017-04-27	2017-05-05	Ongoing		SEVERE	UNLIKELY	Yes
	Multi-organ failure due to prosthetic valve endocarditis (operated on 25/4/17)	Multiple organ dysfunction syndrome	2017-04-27	2017-05-05	2017-05-06	2	SEVERE	NOT RELATED	Yes
	Cardiac tamponade	Cardiac tamponade	2017-04-27	2017-04-28	2017-04-28	1	SEVERE	NOT RELATED	Yes

Table S4

Number of patients that did not reach target volume tube feed including reason for each single day

		Total (N = 26)	*PF (N = 13)*	*SF (N = 13)*
Day 1: n (%)[1]		26 (100.0%)	13 (100.0%)	13 (100.0%)
	No study product administered, n (%)[2]	0 (0.0%)	0 (0.0%)	0 (0.0%)
	Start-up period, n (%)[2]	25 (96.2%)	13 (100.0%)	12 (92.3%)
	Symptoms of intolerance, n (%)[2]	0 (0.0%)	0 (0.0%)	0 (0.0%)
	Medical investigation, n (%)[2]	0 (0.0%)	0 (0.0%)	0 (0.0%)
	Energy intake via other routes, n (%)[2]	2 (7.7%)	1 (7.7%)	1 (7.7%)
	Other, n (%)[2]	1 (3.8%)	0 (0.0%)	1 (7.7%)
Day 2: n (%)[1]		22 (84.6%)	12 (92.3%)	10 (76.9%)
	No study product administered, n (%)[2]	0 (0.0%)	0 (0.0%)	0 (0.0%)
	Start-up period, n (%)[2]	3 (13.6%)	1 (8.3%)	2 (20.0%)
	Symptoms of intolerance, n (%)[2]	1 (4.5%)	1 (8.3%)	0 (0.0%)
	Medical investigation, n (%)[2]	5 (22.7%)	3 (25.0%)	2 (20.0%)
	Energy intake via other routes, n (%)[2]	1 (4.5%)	1 (8.3%)	0 (0.0%)
	Other, n (%)[2]	13 (59.1%)	7 (58.3%)	6 (60.0%)
Day 3: n (%)[1]		19 (73.1%)	10 (76.9%)	9 (69.2%)
	No study product administered, n (%)[2]	0 (0.0%)	0 (0.0%)	0 (0.0%)
	Start-up period, n (%)[2]	0 (0.0%)	0 (0.0%)	0 (0.0%)
	Symptoms of intolerance, n (%)[2]	2 (10.5%)	1 (10.0%)	1 (11.1%)
	Medical investigation, n (%)[2]	4 (21.1%)	3 (30.0%)	1 (11.1%)
	Energy intake via other routes, n (%)[2]	3 (15.8%)	3 (30.0%)	0 (0.0%)
	Other, n (%)[2]	13 (68.4%)	5 (50.0%)	8 (88.9%)
Day 4: n (%)[1]		19 (73.1%)	10 (76.9%)	9 (69.2%)
	No study product administered, n (%)[2]	0 (0.0%)	0 (0.0%)	0 (0.0%)
	Start-up period, n (%)[2]	0 (0.0%)	0 (0.0%)	0 (0.0%)
	Symptoms of intolerance, n (%)[2]	3 (15.8%)	2 (20.0%)	1 (11.1%)
	Medical investigation, n (%)[2]	3 (15.8%)	2 (20.0%)	1 (11.1%)
	Energy intake via other routes, n (%)[2]	4 (21.1%)	3 (30.0%)	1 (11.1%)
	Other, n (%)[2]	13 (68.4%)	6 (60.0%)	7 (77.8%)
Day 5: n (%)[1]		20 (76.9%)	10 (76.9%)	10 (76.9%)
	No study product administered, n (%)[2]	0 (0.0%)	0 (0.0%)	0 (0.0%)
	Start-up period, n (%)[2]	1 (5.0%)	1 (10.0%)	0 (0.0%)
	Symptoms of intolerance, n (%)[2]	3 (15.0%)	2 (20.0%)	1 (10.0%)
	Medical investigation, n (%)[2]	3 (15.0%)	1 (10.0%)	2 (20.0%)
	Energy intake via other routes, n (%)[2]	3 (15.0%)	2 (20.0%)	1 (10.0%)
	Other, n (%)[2]	12 (60.0%)	6 (60.0%)	6 (60.0%)

		Total (N = 26)	*PF (N = 13)*	*SF (N = 13)*
Day 6: n (%)[1]		17 (65.4%)	8 (61.5%)	9 (69.2%)
	No study product administered, n (%)[2]	1 (5.9%)	1 (12.5%)	0 (0.0%)
	Start-up period, n (%)[2]	0 (0.0%)	0 (0.0%)	0 (0.0%)
	Symptoms of intolerance, n (%)[2]	2 (11.8%)	1 (12.5%)	1 (11.1%)
	Medical investigation, n (%)[2]	3 (17.6%)	1 (12.5%)	2 (22.2%)
	Energy intake via other routes, n (%)[2]	2 (11.8%)	1 (12.5%)	1 (11.1%)
	Other, n (%)[2]	10 (58.8%)	4 (50.0%)	6 (66.7%)
Day 7: n (%)[1]		15 (57.7%)	7 (53.8%)	8 (61.5%)
	No study product administered, n (%)[2]	0 (0.0%)	0 (0.0%)	0 (0.0%)
	Start-up period, n (%)[2]	0 (0.0%)	0 (0.0%)	0 (0.0%)
	Symptoms of intolerance, n (%)[2]	0 (0.0%)	0 (0.0%)	0 (0.0%)
	Medical investigation, n (%)[2]	1 (6.7%)	0 (0.0%)	1 (12.5%)
	Energy intake via other routes, n (%)[2]	2 (13.3%)	0 (0.0%)	2 (25.0%)
	Other, n (%)[2]	13 (86.7%)	7 (100.0%)	6 (75.0%)
Day 8: n (%)[1]		14 (53.8%)	5 (38.5%)	9 (69.2%)
	No study product administered, n (%)[2]	0 (0.0%)	0 (0.0%)	0 (0.0%)
	Start-up period, n (%)[2]	0 (0.0%)	0 (0.0%)	0 (0.0%)
	Symptoms of intolerance, n (%)[2]	2 (14.3%)	1 (20.0%)	1 (11.1%)
	Medical investigation, n (%)[2]	2 (14.3%)	0 (0.0%)	2 (22.2%)
	Energy intake via other routes, n (%)[2]	2 (14.3%)	1 (20.0%)	1 (11.1%)
	Other, n (%)[2]	12 (85.7%)	5 (100.0%)	7 (77.8%)
Day 9: n (%)[1]		9 (34.6%)	4 (30.8%)	5 (38.5%)
	No study product administered, n (%)[2]	0 (0.0%)	0 (0.0%)	0 (0.0%)
	Start-up period, n (%)[2]	1 (11.1%)	1 (25.0%)	0 (0.0%)
	Symptoms of intolerance, n (%)[2]	0 (0.0%)	0 (0.0%)	0 (0.0%)
	Medical investigation, n (%)[2]	2 (22.2%)	0 (0.0%)	2 (40.0%)
	Energy intake via other routes, n (%)[2]	1 (11.1%)	1 (25.0%)	0 (0.0%)
	Other, n (%)[2]	7 (77.8%)	4 (100.0%)	3 (60.0%)
Day 10: n (%)[1]		8 (30.8%)	4 (30.8%)	4 (30.8%)
	No study product administered, n (%)[2]	0 (0.0%)	0 (0.0%)	0 (0.0%)
	Start-up period, n (%)[2]	0 (0.0%)	0 (0.0%)	0 (0.0%)
	Symptoms of intolerance, n (%)[2]	1 (12.5%)	0 (0.0%)	1 (25.0%)
	Medical investigation, n (%)[2]	0 (0.0%)	0 (0.0%)	0 (0.0%)
	Energy intake via other routes, n (%)[2]	1 (12.5%)	1 (25.0%)	0 (0.0%)
	Other, n (%)[2]	7 (87.5%)	4 (100.0%)	3 (75.0%)
Day 11: n (%)[1]		9 (34.6%)	4 (30.8%)	5 (38.5%)
	No study product administered, n (%)[2]	0 (0.0%)	0 (0.0%)	0 (0.0%)

	Total (N = 26)	PF (N = 13)	SF (N = 13)
Start-up period, n (%)[2]	0 (0.0%)	0 (0.0%)	0 (0.0%)
Symptoms of intolerance, n (%)[2]	1 (11.1%)	0 (0.0%)	1 (20.0%)
Medical investigation, n (%)[2]	0 (0.0%)	0 (0.0%)	0 (0.0%)
Energy intake via other routes, n (%)[2]	1 (11.1%)	1 (25.0%)	0 (0.0%)
Other, n (%)[2]	8 (88.9%)	4 (100.0%)	4 (80.0%)
Day 12: n (%)[1]	8 (30.8%)	4 (30.8%)	4 (30.8%)
No study product administered, n (%)[2]	0 (0.0%)	0 (0.0%)	0 (0.0%)
Start-up period, n (%)[2]	0 (0.0%)	0 (0.0%)	0 (0.0%)
Symptoms of intolerance, n (%)[2]	3 (37.5%)	2 (50.0%)	1 (25.0%)
Medical investigation, n (%)[2]	2 (25.0%)	1 (25.0%)	1 (25.0%)
Energy intake via other routes, n (%)[2]	1 (12.5%)	1 (25.0%)	0 (0.0%)
Other, n (%)[2]	5 (62.5%)	3 (75.0%)	2 (50.0%)
Day 13: n (%)[1]	7 (26.9%)	3 (23.1%)	4 (30.8%)
No study product administered, n (%)[2]	0 (0.0%)	0 (0.0%)	0 (0.0%)
Start-up period, n (%)[2]	0 (0.0%)	0 (0.0%)	0 (0.0%)
Symptoms of intolerance, n (%)[2]	0 (0.0%)	0 (0.0%)	0 (0.0%)
Medical investigation, n (%)[2]	0 (0.0%)	0 (0.0%)	0 (0.0%)
Energy intake via other routes, n (%)[2]	1 (14.3%)	1 (33.3%)	0 (0.0%)
Other, n (%)[2]	7 (100.0%)	3 (100.0%)	4 (100.0%)
Day 14: n (%)[1]	7 (26.9%)	2 (15.4%)	5 (38.5%)
No study product administered, n (%)[2]	0 (0.0%)	0 (0.0%)	0 (0.0%)
Start-up period, n (%)[2]	0 (0.0%)	0 (0.0%)	0 (0.0%)
Symptoms of intolerance, n (%)[2]	0 (0.0%)	0 (0.0%)	0 (0.0%)
Medical investigation, n (%)[2]	0 (0.0%)	0 (0.0%)	0 (0.0%)
Energy intake via other routes, n (%)[2]	4 (57.1%)	1 (50.0%)	3 (60.0%)
Other, n (%)[2]	5 (71.4%)	2 (100.0%)	3 (60.0%)

Table S5

Summary statistics for ASAT (U/L) day 5

		Statistic	*Total (N = 26)*	*New peptide formula (N = 13)*	*Standard formula (N = 13)*	*P-value¹*
Number of subjects (N)		n	24	12	12	
ASAT (U/L), outside normal range	No	n (%)	10 (47.6%)	5 (45.5%)	5 (50.0%)	
	Yes	n (%)	11 (52.4%)	6 (54.5%)	5 (50.0%)	
	Missing	n	3	1	2	
ASAT (U/L), if outside normal range, clinically relevant	No	n (%)	0 (0.0%)	0 (0.0%)	0 (0.0%)	
	Yes	n (%)	11 (100.0%)	6 (100.0%)	5 (100.0%)	
ASAT (U/L), if clinically relevant, related to underlying condition	No	n (%)	0 (0.0%)	0 (0.0%)	0 (0.0%)	
	Yes	n (%)	11 (100.0%)	6 (100.0%)	5 (100.0%)	
ASAT (U/L)		n (Nmiss)	21 (3)	11 (1)	10 (2)	
		Mean (SD)	65.0 (51.1)	79.6 (64.6)	48.9 (24.9)	
		Median (Q1-Q3)	43 (33-86)	55 (33-126)	42 (29-71)	0.364
		Min-Max	20-221	20-221	22-91	
		95% confidence interval	41.7-88.3	36.2-123.1	31.1-66.7	

(N): Number of subjects of the analysis population.
¹P-value is based on a stratified Mann-Whitney test (van Elteren test) with site as stratification factor.

Table S6 Number of patients (n) per intervention day

Day	***PF*** *(n)*	***SF*** *(n)*
1	13	13
2	13	13
3	13	13
4	12	12
5	10	11
6	8	11
7	7	11
8	5	11
9	4	7
10	4	6
11	4	6
12	4	6
13	3	6
14	2	6
